# Estimation of seroprevalence of HIV, hepatitis B and C virus and syphilis among blood donors in the hospital of Aïoun, Mauritania

**DOI:** 10.11604/pamj.2017.28.118.12465

**Published:** 2017-10-06

**Authors:** Boushab Mohamed Boushab, Ould Cheikh Melaïnine Mohamed Limame, Fall-Malick Fatim Zahra, Savadogo Mamoudou, Belizaire Marie Roseline Darnycka, Sow Mamadou Saliou

**Affiliations:** 1 Department of Internal Medicine, Aïoun Regional Hospital, Hodh El Gharbi, Mauritania; 2 Ambulatory Treatment Center, National Hospital Center of Nouakchott, Mauritanie; 3 National Institute of Hepatology-Virology in Nouakchott, School of Medicine, Nouakchott, Mauritania; 4 Department of Infectious Diseases, University Teaching Hospital Yalgado Ouédrago, Ouagadougou, Burkina Faso; 5 Health Security and Emergency Officer, WHO World Health Emergency Programme, Mauritania; 6 Department of Infectious Diseases, University Teaching Hospital Donka, Conakry, Guinée

**Keywords:** Seroprevalence, HIV, hepatitis B, hepatitis C, syphilis, blood donors, Aïoun? Mauritania

## Abstract

**Introduction:**

To estimating the seroprevalence of HIV, hepatitis B, hepatitis C and syphilis among blood donors in the Aïoun hospital.

**Methods:**

This is a retrospective study from 1 January 2010 to 31 December 2015.

**Results:**

On the five-year study period, 1,123 donors were collected. Of these, 182 were HIV-positive, an overall prevalence of 16.2% with predominance in male with a sex ratio Man/Woman of 5.2. The average age of donors was 32.7 ± 10 years (range 17-73 years). The most represented that age group 21-30 years (40.5%). The seroprevalence found were 1.2% for HIV, 11.8% for HBV, HCV 0.2% and 3% for syphilis. Co-infection was found in 0.7% of which 0.5% of dual HIV HBV/Syphilis and 0.2% in HBV/HIV.

**Conclusion:**

The transmission of infectious agents related to transfusion represents the greatest threat to transfusion safety of the recipient. Therefore, a rigorous selection and screening of blood donors are highly recommended to ensure blood safety for the recipient.

## Introduction

Blood transfusion is a medical therapeutic act [1–3]. However, despite the benefits, each patient is transfused at risk for transfusion-transmissible infections, mainly HIV, hepatitis B (HBV), hepatitis C (HCV) and *Trepanoma pallidum* (*T. pallidum*) [2, 3]. The morbidity and mortality resulting from transfusion have serious consequences, not only for the beneficiaries themselves but also for their families, their communities and society in general [3, 4]. Studies conducted in sub-Saharan Africa show that there is a high prevalence of these infections [3–12]. In Mauritania, studies of prevalence among blood donors held in Nouakchott in 1999, 2000 and 2009 showed respective HCV seroprevalence of 1.1% and 2.7% [11, 13] HBV and 15.3% and 20.3% [11, 14]. This study has aimed to update the seroprevalence data of 4 serological markers (HIV, HBV, HCV, anti-Ag-*Trepanoma pallidum*) tested in blood donors from the hospital Aïoun, in accordance with national strategy for patient safety.

## Methods

This is a retrospective descriptive study among blood donors in the regional hospital Aïoun, Hodh El Gharbi (Mauritania), over a period of 5 years from January 1 2010 to 31 December 2015. This hospital is the reference center of the Hodh El Gharbi region (Mauritania) and welcomes an urban and rural population. Aïoun el Atrouss (62 984 inhabitants) is the administrative capital of the wilaya of Hodh El Gharbi (288,772 inhabitants). Wilaya is located 800km from Nouakchott (capital) South-East of the country and has the only regional hospital specific reference to medical care and/or surgery which offers the public a range of treatments specifically in the areas of dentistry, general medicine, surgery, obstetrics and ophthalmology. Donors were either volunteers or relatives or friends of patients apparently healthy weighing 50kg or more with a hemoglobin > 12.5 g/dl. Before every donation, sorting through a donor questionnaire stage, a complete physical examination, serologic screening of the major transfusion transmissible infections and ABO grouping. The confidentiality of donors was met, as the anonymity of the gift obliges. No information revealing their identity was collected in this study. The parameters studied were sex, age, serology for HIV, HBV, HCV and syphilis. Mark of HBV HBsAg was performed using an immunochromatographic test, Determine™ HBsAg Test (AlereMedical Co. Ltd, Japan). The demonstration of antibodies to HIV and those anti-HCV-Ab were carried out respectively by the test, Determine™ HIV-1/2 (Alere Medical Co. Lt, Japan) ant the Rapid test SignalMT HCV Serum/Plasma Dipstrip Test for the hepatite C (Alerehealthcare, South Africa). Seropositivity for syphilis in turn uses a completely screening by a Rapid-Plasma-Réagin test (syphilis RPR test, Human Gesellschaft für Biochemicaund Diagnostic amb H, Germany) then the positive samples were passed to the TPHA (*Treponema Pallidum Hemagglutination Assay*) and the Venereal Disease Research Laboratory (VDRL), for confirmation. Entry and data analysis were performed using Epi Info version 6.4 software. For the comparison of quantitative variables the Chi-square test was used. A p value < 0.05 was selected as the significance threshold.

## Results

Over the study period of 5 years, in 1123 donors were collected. The male was predominant, with a ratio sex- Male/Female 5.2. The average age of donors was 32.7 ± 10 years (range 17-73 years). Up to 20 years old and the age group 21 to 30 years, respectively represented 11% and 40.5% of donors. The age group of 31 to 40 accounted for 26.8% and the 41 to 50 years 14.7%. The age group 51 and older accounted for 7% of donors. Considering all the markers, 182 among the donors presented seropositivity, an overall prevalence of 16.2%. The prevalence of HIV, HBV, HCV and syphilis were observed in 1.2%, 11.8%, 0.2% and 3%. Co-infection was found in 0.7% of cases and 0.5% of dual-infection HBV/Syphilis and 0.2% in the double HBV/HIV infection. A statistically significant difference was observed between HBV carriage and the most affected age group (p = 0.009) and between syphilis and age groups (p = 0.02) (Table 1).

**Table 1:** Comparing age groups of infected and non-infected blood donors

Age class	Blood donors	HBV	HCV	HIV	*Treponema pallidum*
<20	123	15	0	0	7
21-30	455	63	0	4	19
31-40	301	36	1	6	7
41-50	165	6	0	1	0
>51	79	12	1	3	1
Total	1123	132	2	14	34
*p*		0.009	0.13	0.08	0.02

## Discussion

The findings of this study reflect a general idea about the prevalence of infectious markers in a rural hospital with very limited means. The results can therefore only be interpreted within these limits. However, they highlight a greater representation of men with a sex-ratio Male/Female 5.1. This male predominance may be explained by socio-cultural markers making man the ideal candidate to for blood donation. On gynéco of obstetric physiological factors such as menstrual cycles, pregnancy, breastfeeding can also reinforce this trend. These factors may indeed encourage many women to not donate blood [15]. This male was already provided by other African writers in Nigeria, Mali, Niger, Ivory Coast and Cameroon [4–6, 16, 17]. The average age of our donors was similar to that reported by other African studies in Mali, Nigeria and Cameroon [4, 5, 17]. In our study, the overall prevalence of biomarkers studied in blood donors was 16.2%. This percentage is lower than that reported by other African writers in Burkina Faso, Nigeria, Niger, Cameroon and Tanzania [3, 4, 6, 7, 9]. The most represented age group was the 21 to 30 years. These results are similar to those in other african study [3, 8, 9, 18, 19]. HIV seroprevalence reported as part of this study was 11.8%. This proportion was lower than previously reported in studies conducted in Nouakchott in 1999 and 2012 [11, 14]. It was also lower than those made in the african sub-region, including Mali [20], in Sénégal [21], in Burkina Faso [9], in Niger [6], Ivory Coast [16] and in Nigeria [4] but was higher than those made in Morocco [22], in Ethiopia [8], in Tanzania [7], Democratic Republic of Congo (RDC) [10] and Cameroon [3, 17]. HVC, the prevalence was 0.2%.

This figure was lower than previously found in a study conducted in 1999 and 2007 in Nouakchott [11, 13], as well as those reported in studies African countries [3, 4, 6–9, 17, 20, 22–26]. In our study, HIV seroprevalence was 1.2%. These figures are higher than the estimated national prevalence was 0.4% [27]. This prevalence is higher than those made in Morocco and Algeria [28, 29]. As against it remains lower than those reported in Mali, Burkina Faso, Niger, Ivory Coast, Nigeria, Ethiopia, Tanzania, DRC and Cameroon [3, 4, 6–10, 16, 17, 20]. The prevalence of syphilis was 3%. These figures are lower than those found in other studies in Africa, including Burkina Faso, Tanzania, DRC and Cameroon [3, 8–10, 17] but it remains higher than those reported in Mali, Niger, Nigeria and Ethiopia [4, 6, 8, 20]. As regards co-infections, associations HBV/HIV and HBV/syphilis were observed in respectively 0.2% and 0.5% of cases. As indiquépar other studies, this association could be due to the fact that these infections share similar transmission, mainly blood and sexual behavior at high risk of infection [4, 8, 30]. Other studies have shown an association between HIV and syphilis, probably because of their sexual mode of transmission similaireet especially that the mucocutaneous lesions caused by syphilis is a gateway to HIV infection [8, 31]. In our study the seroprevalence of HIV, HBV and syphilis were the highest in the different age groups in comparison with other markers (HCV and syphilis studied). It y'avait statistically significant differences between seropositivity to HBV and syphilis and sex and age. These results may indicate certain risk behaviors of higher infection in men, such as multiple sexual partnerships, etc, as it could also be linked to a lower representation of female blood donation. This difference can be attributed to differences in methodologies, in fact the others have worked on the different categories of donors whereas in our study, it is only for family donors. Furthermore, in terms of the reagents used for serology, some authors have adopted confirmatory tests, plus tests used in this study.

## Conclusion

Despite enormous progress in the framework of transfusion safety, blood transfusion is a medical therapeutic act which exposes the recipient to a risk of contamination by infectious agents transmissible through blood. Therefore, to enhance good blood safety for the recipient, it is necessary to focus on a rigorous selection and retention of donors on the one hand and the use of screening méthods standards minimizing the window period. Furthermore, studies on the residual risk to measure the likelihood of transmission of various infectious agents by blood products, are entirely justified, especially for HBV, which is a real public health problem in our context, with prevalence approaching 20% in different groups (surveys conducted among different groups between 2007 and 2009).

### What is known about this topic

- Blood transfusion is a medical therapeutic act;
- Each patient is transfused at risk for transfusion-transmissible infections If the blood is not secured;
- The morbidity and mortality resulting from transfusion have serious consequences for patients, communities and society in general.

### What this study adds

- To our knowledge, this study is the first in the country to study these 4 markers at the same time;
- Estimating the seroprevalence of infectious markers in blood donors;
- To strengthen transfusion safety in recipients since there are only intra-family donors in most cases.

## Competing interests

The authors declare no competing interests.
